# Maternal Stress, Familial Ties, and Children’s Well-Being Across 23 Years

**DOI:** 10.1093/geroni/igab046.2248

**Published:** 2021-12-17

**Authors:** Jennifer Cleary, Jasmine Manalel, Simon Brauer

**Affiliations:** 1 University of Michigan, Ann Arbor, Michigan, United States; 2 National Human Genome Research Institute, Bethesda, Maryland, United States

## Abstract

According to the family stress model, parental stress impacts child well-being through several mechanisms, which may be amplified in ethnic/racial minority families given increased experiences of stress. We extend this model to examine associations between maternal stress and child well-being at three points spanning 23 years, beginning when children were aged 8-12 years and mothers were aged 24-59 (n=193 dyads). Preliminary results indicate that maternal stressors are associated with increased depressive symptoms in childhood (B=3.56, p<0.001), and this association was stronger among Black children compared to White (B=4.12, p<0.001). Effects of maternal stress on children’s depressive symptoms strengthened among White children with proportionally larger kin networks (B=0.05, p<0.001). However, this association weakens as children enter adulthood. Future work will focus on identifying social resources that account for changes in the intergenerational effects of stress.

